# Complete genome of *Priestia filamentosa* H146 isolated from tobacco leaves

**DOI:** 10.1128/mra.00195-24

**Published:** 2024-07-25

**Authors:** Qin Gao, Mengmeng Yang, Yuan Ji, Liwei Hu, Xiangzhou Dong, Taibo Liang, Zhen Zhai, Yanming Cheng, Huaxin Dai, Limei Zhao, Guo Zhang, Qifa Zhou

**Affiliations:** 1 Anhui Wannan Tobacco Co., Ltd., Xuancheng, Anhui, China; 2 Zhengzhou Tobacco Research Institute of CNTC, Zhengzhou, Henan, China; 3 Shandong Institute for Food and Drug Control, Jinan, Shandong, China; University of Strathclyde, Glasgow, United Kingdom

**Keywords:** *Priestia filamentosa*, tobacco, genome sequencing, carbohydrate active enzymes

## Abstract

We report the complete genome of *Priestia filamentosa* H146 isolated from tobacco leaves. H146 contained a circular chromosome and five circular plasmids. A total of 4,669 genes were predicted, of which 4,372 genes were in the chromosome and other genes were located on plasmids. The genome sequence data provide an important basis for studying *Priestia filamentosa*.

## ANNOUNCEMENT

*Priestia* is a genus of mostly Gram-positive rod-shaped bacteria in the Bacillaceae family (1 – 3), which has shown significant beneficial roles in various plant species that are enacted via diverse mechanisms (2 – 5). We isolated a new strain of *Priestia filamentosa* H146 from leaves of cigar variety Dexue 1 from Deyang in Sichuan province in China. A single leaf from a single plant was surface sterilized by 75% ethanol and divided into many 5 × 5 mm blocks; 5  g of blocks was added to a 45-mL-sterilized phosphate solution for washing at 180  r/min for 2  h. Then, the solution was diluted to 10^−3^ and spread on the starch agar plate (peptone 10 g/L, beef extract 3 g/L, sodium chloride 5 g/L, soluble starch 3g/L, agar 20g/L, pH 7.0) (6, 7), which was incubated at 37°C for 36 h-48h in aerobic condition until the isolates formed colonies. A pure colony was obtained by restreaking three times, which was then stored in the laboratory.

H146 strain was cultured in NA medium (peptone 10 g/L, beef extract 3 g/L, sodium chloride 5 g/L, pH 7.0) for shaking at 37°C with 180 rpm for 24 h. Genomic DNA was extracted from the same colony for both PacBio and Illumina sequencing by using DNeasy UltraClean/NoviPure Microbial Kits (QIAGEN, Germany). Genomic DNA was sheared into selected fragment sizes ranging from 6 kb to 20 kb by using the Covaris g-TUBE device (Woburn, USA) and size-selected by blue pippin fragment (Sage Science, USA). Libraries for single-molecule real-time sequencing were constructed, with insert size of 10 kb using the SMRT bell TM Template kit (Pacific Biosciences, USA), then sequenced using PacBio Sequel II (Pacific Biosciences, USA) in Beijing Novogene Co., Ltd. in China. 1 µg of genomic DNA was used for the construction of sequencing libraries with NEBNext Ultra DNA Library Prep Kit (NEB, USA) and sequenced by NovaSeq PE150. PacBio reads were used for assembly, and Illumina reads were only used for correction.

The Illumina read quality was controlled by Trimmomatic (version: 0.39) (8), and the reads with adapter sequences (overlap ≥15 bp), low-quality (quality value ≤38, overlap ≥40%), and a higher rate of N (>10%) were abandoned. All reads of Pacbio sequencing were used for assembly; *de novo* genome assembly was done by Canu software (version：2.0) with setting -d run1 -p H146 genome size = 4.5 m pacbio subreads.fastq.gz, which was followed by an error correction with default parameters by three rounds of Racon (version 1.4.13) with Pacbio sequencing reads and three rounds of Pilon version 1.22 with Illumina sequencing reads (9 – 11). Unless otherwise noted, default parameters were used for all software. If the 3′ end and 5′ end of assembled chromosome have overlapped with more than 2 k, it is considered to be looped; and the overlapping part will be removed. Resulting circular replicon sequences of chromosomes by Canu were rotated to *dnaA* gene.

Strain H146 contained a circular chromosome and five circular plasmids (Table 1). Chromosome size was 4,270,366 bp, and plasmid sizes vary from 7,599 bp to 109,609 bp. NCBI Prokaryotic Genome Annotation Pipeline (Version 6.6) (12) predicted 4,669 genes (Table 1). Digital DNA-DNA hybridization (dDDH) analysis (13) revealed that H146 genome sequence CP136435 has a high identity (98.18%) with *Priestia filamentosa* IG3 (GCA_032027705.1).

**TABLE 1:** Genome features of *P. filamentosa* H146 strain

Feature	Value
Sum of all Illumina read lengths (Mb)	1,917
Number of Illumina reads	12,782,446
Sum of all Pacbio read lengths (Mb)	2,713
Number of Pacbio reads	608,023
N50 Pacbio read length (bp)	6,183
Genome size (bp)	4,573,697
GC content in genome (%)	36.5
Gene number	4,669
Gene length/genome (%)	80.3
# of tRNAs	47
# of ncRNAs	5
Chr1 size (genes)	4,270,366 (4,372)
Chr1 NCBI accession	CP136435
GC content in Chr1 (%)	36.59
Plasmid p1 size (genes)	109,609 (114)
Plasmid p1 NCBI accession	CP136436
GC content in plasmid p1 (%)	34.43
Plasmid p2 size (genes)	7,599 (5)
Plasmid p2 NCBI accession	CP136437
GC content in plasmid p2 (%)	33.83
Plasmid p3 size (genes)	79,564 (81)
Plasmid p3 NCBI accession	CP136438
GC content in plasmid p3 (%)	34.98
Plasmid p4 size (genes)	8,279 (13)
Plasmid p4 NCBI accession	CP136439
GC content in plasmid p4 (%)	36.06
Plasmid p5 size (genes)	98,280 (84)
Plasmid p5 NCBI accession	CP136440
GC content in plasmid p5 (%)	34.77

## Data Availability

The raw Pacbio and Illumina reads were deposited to NCBI SRA with SRR26533013 and SRR27054199 as accession numbers, respectively. The chromosome and five plasmid sequences of *P. filamentosus* H146 were submitted to NCBI with CP136435, CP136436, CP136437, CP136438, CP136439, and CP136440 as accession numbers, which were under the BioProject ID PRJNA1025957 and the Biosample accession number SAMN37731258.
